# EPIDEMIOLOGY, CLINICAL MANIFESTATIONS, AND DIAGNOSIS OF CHIKUNGUNYA FEVER: LESSONS LEARNED FROM THE RE-EMERGING EPIDEMIC

**DOI:** 10.4103/0019-5154.60355

**Published:** 2010

**Authors:** Alladi Mohan, DHN Kiran, I Chiranjeevi Manohar, D Prabath Kumar

**Affiliations:** *From the Department of Medicine, Sri Venkateswara Institute of Medical Sciences, Tirupati - 517 507, India.*

**Keywords:** *Chikungunya*, *epidemiology*, *diagnosis*, *treatment*

## Abstract

Chikungunya fever, caused by “Chikungunya virus,” is an arbovirus disease transmitted by the bite of infected mosquitoes belonging to the genus *Aedes*. Chikungunya fever epidemics have been reported from several countries around the world. The disease that was silent for nearly 32 years re-emerged in the October 2005 outbreak in India that is still ongoing. The incubation period ranges from 3 to 12 days. The onset is usually abrupt and the acute stage is characterized by sudden onset with high-grade fever, severe arthralgias, myalgias, and skin rash. Swollen tender joints and crippling arthritis are usually evident. In the chronic stage, relapses that include sensation of fever, asthenia, exacerbation of arthralgias, inflammatory polyarthritis, and stiffness may be evident. Neurological, ocular, and mucocutaneous manifestations have also been described. Chronic arthritis may develop in about 15% of the patients. Viral culture is the gold standard for the diagnosis of Chikungunya fever. Reverse transcription polymerase chain reaction and real-time loop-mediated isothermal amplification have also been found to be useful. Serodiagnostic methods for the detection of immunoglobulin M and immunoglobulin G antibodies against Chikungunya virus are more frequently used. Chikungunya is a self-limiting disease; however, severe manifestations such as meningoencephalitis, fulminant hepatitis, and bleeding manifestations may sometimes be life-threatening. Treatment is symptomatic and supportive. Prevention by educating the community and public health officials, vector control measures appear to be the best approach at controlling Chikungunya fever as no commercially available vaccine is available for public use in India for this condition presently.

## Introduction

Chikungunya fever is an important arthropod-borne virus (arbovirus) disease.[1–3] Until recently, Chikungunya fever attracted only minor interest in the medical community and did not evoke the fear associated with other arboviruses, such as dengue and West Nile viruses. The recent resurgence of Chikungunya fever has drawn global attention due to its explosive onset, rapid spread, high morbidity, and myriad clinical manifestations.[4–8] From 2006 onward, Chikungunya fever has emerged, even in nonendemic areas as an important disease in returning travelers. Indeed, travelers have emerged as sentinels, transporters, and transmitters of the disease.[4,7] The social and economic impact of Chikungunya fever has also been tremendous especially in India.[1,9]

## Historical Background

There are historical accounts of epidemics of fever, arthralgias/arthritis, and rash, resembling what is now called “Chikungunya fever” dating back to 1824 from India and elsewhere.[10] Chikungunya fever was first described in 1952,[11,12] following an outbreak on the *Makonde* Plateau, along the border between Tanganyika and Mozambique.

## Etymology and History of the Name “Chikungunya”

For a long time, it was erroneously reported that the word “Chikungunya” was derived from the “Swahili” language both in lay press and the media and reputed medical journals.[13] The word “Chikungunya” is derived from the *Makonde* language, spoken by an ethnic group in south-east Tanzania and northern Mozambique from the root verb “kungunyala”, meaning “to dry up or become contorted,” and signifies the cause of a contortion or folding.[12,14] Literally, the word “Chikungunya” translates to “that which bends up” in reference to the stooped posture developed due to the rheumatological manifestations of the disease. In Congo, it has been called “buka-buka”, meaning “broken-broken” once again reflecting the incapacitating arthralgias that are common acute manifestations of Chikungunya fever.[15]

## Pathogenesis

### Natural history and transmission

Chikungunya fever epidemics display secular, cyclical, and seasonal trends. These epidemics are characterized by explosive outbreaks interspersed by periods of disappearance ranging from several years to a few decades. The exact reason for this behavior still remains a mystery. Several mechanisms including a complex interaction between various factors such as the susceptibility of humans and the mosquito vectors to the virus; conditions facilitating mosquito breeding resulting in a high vector density, ability of the vector to efficiently transmit the virus are all thought to play a role.[1,4,7] International travel has facilitated the introduction of the virus from endemic areas to other areas resulting in outbreaks of the illness, and imported cases Chikungunya fever have been documented in France, Italy, Australia, and United States of America also.[4,16–18]

The natural cycle of the virus is human-mosquito-human; evidence is available regarding the existence of epizootic cycles that may maintain the virus during the interepidemic period.[1,4–8,19] During epidemics, human beings serve as the Chikungunya virus reservoirs; during interepidemic periods, several vertebrates, such as monkeys, rodents, birds, have been implied as the reservoir. Little is known regarding whether and how the Chikungunya virus is maintained in the wild in Asia. Unlike dengue virus, there is no evidence for transovarial transmission of Chikungunya virus in mosquitoes. Variations in the geographical strains of *Aedes* mosquitoes regarding their susceptibility to infection and ability to transmit the virus may be crucial factors in determining endemicity of Chikungunya virus in a given region.[1,4–8,19] Vertical maternal-fetal transmission has been documented in pregnant women affected by Chikungunya fever.[20]

### The virus

The virus causing Chikungunya fever is an alphavirus of the family Togaviridae. It has a genome consisting of a linear, positive-sense, approximately 11.8 kb single stranded ribonucleic acid (RNA) molecule, a 60-70 nm diameter capsid and a phospholipid envelope.[4,7,8] Studies on Chikungunya virus isolates collected from various geographical areas indicate that three lineages with distinct genotypic and antigenic characteristics exist. These include the West-African; the East, Central, Southern African (ECSA) phylogroups that have contributed to epidemics in Africa; and the Asian phylogroup. Phylogenic analysis based on partial sequences of *NS4* and *E1* genes showed that isolates from India during the present epidemic and the isolates from the ongoing Indian Ocean outbreak represent a distinct clade within the ECSA phylogroup,[1,21–23] while all earlier isolates from India (1963 to 1973) were Asian genotype.[24–30]

### Mutations in the Chikungunya virus genome and infectivity

A mutation at residue 226 of the membrane fusion glycoprotein E1 (E1-A226V) was detected in more than 90% of Chikungunya virus isolates from Reunion Islands after September, 2005. By reducing the cholesterol dependence of the virus to infect mosquito hosts, this mutation is postulated to have facilitated the replication and transmission of the virus by this mosquito.[31,32] This rapid pathogen adaptation to ecologic perturbation appears to be a unique example of the “evolutionary convergence” occurring in nature.[33]

### Molecular pathogenesis

Little information is available on the molecular pathogenesis of Chikungunya fever. Given the similarities between the two, molecular pathogenesis of Chikungunya fever may be similar to the observations made on the molecular and cellular aspects of Ross River virus (RRV) that also causes epidemics of fever, polyarthritis (including rheumatic symptoms), and mucocutaneous manifestations similar to that seen with Chikungunya fever.[34] A defective cell-mediated immune response (CMI) where CD8+ T-lymphocytes are absent or inactive has been thought to be the cause of chronic disease and viral persistence. Oversecretion of toxic chemokines or apoptosis are postulated as causes of cell/tissue destruction and associated clinical symptoms.[8] An antibody-dependent enhancement (ADE) mechanism similar to that suggested for dengue viruses[35] has also been implicated in the pathogenesis. Cell tropism of Chikungunya virus in the murine brain; susceptibility of human adherent cells (epithelial and endothelial cells, primary fibroblasts, and macrophages) to Chikungunya virus whose replication induces a cytopathic effect and apoptosis;[4,36] are all leads that need to be studied in greater detail. Presence of viral antigen in muscle progenitor cells (also called muscle satellite cells) in a muscle biopsy from a patient who had a clinical relapse some weeks after disease onset[4,37] suggests the possibility of persistence of Chikungunya virus in some patients long after initial viremia. Further research is required in this area to clarify these issues.

### The vector

In Asia and the Indian Ocean region, Chikungunya fever is transmitted by the bite of mosquitoes of the genus *Aedes* (which also transmit the dengue virus). *Aedes aegypti* is considered to be the principal vector and *A. albopictus* (Asian Tiger mosquito) has also recently emerged as an important vector. *A. aegypti* predominantly breeds in stored fresh water, such as desert coolers, flower vases, water-tanks, etc., and in peri-domestic areas (discarded household junk items like vehicular tyres, coconut shells, pots, cans, bins, etc.,) in urban and semiurban environments.[38,39] Adult mosquitoes rest in cool and shady areas and bite humans during the daytime. Insecticide treated bed nets are, therefore, of limited use against *Aedes* mosquitoes. The bite of only the female mosquito is considered to be infective because a blood meal is required for the formation of the egg.

The same vectors can sometimes transmit several arboviruses, and mixed epidemics of Chikungunya fever and dengue fever;[7,40] Chikungunya fever, dengue fever, and *Plasmodium falciparum* malaria[41] have sometimes been documented.

## Epidemiology

### Global scenario

Following the report from Tanganyika in 1952,[11,12] Chikungunya epidemics have been reported from several parts of the world including Africa, Asia, and elsewhere. In South-East Asia, epidemics have been documented in India, Pakistan, Sri Lanka, Myanmar, Thailand, Indonesia, the Philippines, Cambodia, Vietnam, Hong Kong, and Malaysia.[1,7,38,42] Since 2003, there have been outbreaks in the islands of the Pacific Ocean, including Madagascar, Comoros, Mayotte the Seychelles, and Mauritius, and the Reunion Island (French overseas district in the Indian Ocean).[42]

### India

Since the first Indian report from Kolkata (Calcutta then),[24] several outbreaks of Chikungunya fever have been documented from different parts of India including Vellore,[25] Chennai (then called Madras) in Tamil Nadu, and Puducherry (then called Pondicherry)[26], Visakhapatnam, Rajahmundry, and Kakinada in Andhra Pradesh,[1,7,21,27,38] Nagpur,[28] and Barsi[29] in Maharastra. Occasional cases were recorded in Maharastra State between 1983 and 2000.[30] Keeping with the character of the disease, Chikungunya fever has re- emerged in India after nearly 32 years in October 2005[1,7,38] and the outbreak is ongoing.

In the investigation carried out by the National Institute of Virology[21] from several districts in Andhra Pradesh, Karnataka, and Maharastra (*n* = 1938), the occurrence of Chikungunya epidemic was confirmed. During the current outbreak as per the current release by the Ministry of Health and Family Welfare, Government of India, 13,91,165 cases suspected to be Chikungunya fever have been recorded from several parts of India during the year 2006.[1,38] The corresponding figures for the subsequent years have been 59,535 cases (2007) and 71,222 (till November 2008),[1,38] and 13,117 (till June 2009).[43] The current status of Chikungunya fever in various parts of India is shown in Table 1.[43] Some workers have approximated that these figures are underestimates and the actual figures could be up to five times these numbers.[44]

**Table 1:** State-wise suspected Chikungunya cases in India

State	2009 till June
Karnataka	9,816
Andhra Pradesh	549
Tamil Nadu	971
Kerala	701
Goa	77
West Bengal	127
Maharashtra	291
Gujarat	511
Rajasthan	74
Total	13,117

## Clinical Manifestations

### Acute stage

Chikungunya fever affects all age groups, and both genders are equally affected. The incubation period ranges from 3 to 12 days (usually 3-7 days).[1,4–8] In susceptible populations, the attack rates can be as high as 40-85%.

### Onset

Prodromal symptoms are rare. In the acute stage, the onset is usually abrupt and sudden with high-grade fever (usually 102-105 °F), severe arthralgias, myalgias, and skin rash.[1,4–8] Headache, throat discomfort, abdominal pain, and constipation may also be evident. Conjunctival suffusion, persistent conjunctivitis, cervical, or sometimes generalized lymphadenopathy may be present.

## Mucocutanoeus Manifestations

Several mucocutaneous manifestations, such as morbilliform eruption, scaling, macular erythema, intertrigo, hypermelanosis, xerosis, excoriated papules, urticaria, and petechial spots have been described in patients with Chikungunya fever.[45,46] These are described in detail elsewhere in this issue of the journal.

### Arthropathy

**Figure 1:**
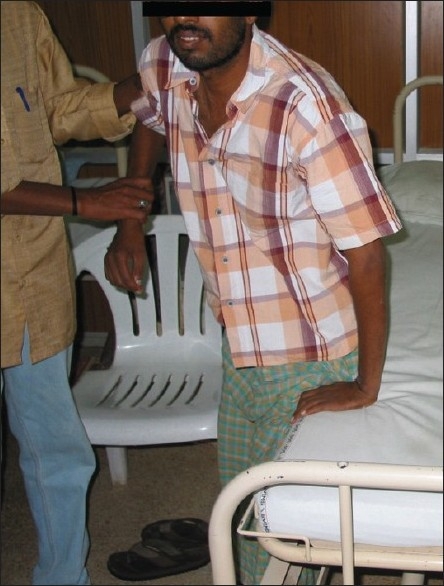
Crippling arthritis involving small joints of the hands, wrist, elbow, shoulder, knee, and ankle joints in a patient with Chikungunya fever. As the name “Chikungunya” (“that which bends up”) suggests, stooped posture of the patient is evident

**Figure 2:**
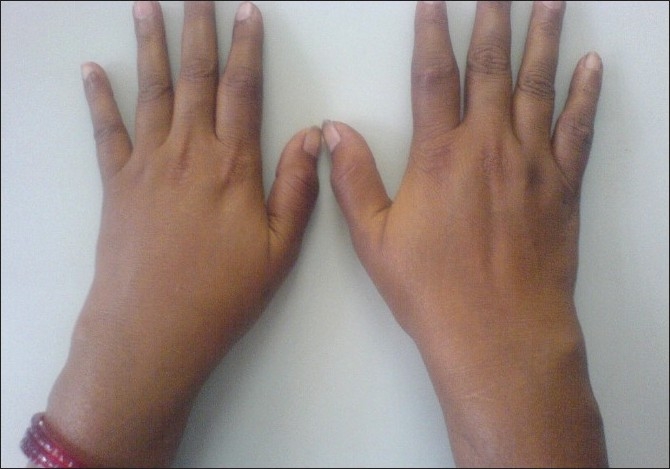
Symmetrical inflammatory polyarthritis of the small joints of the hands and tenosynovitis of the wrist joints in a patient with chronic stage of Chikungunya fever

The Chikungunya viral polyarthropathy frequently involves the small joints of the hand, wrist, and ankles and the larger joints such as knee and shoulder; more than 10 joint groups may be involved.[4–8,47] Axial involvement is common. The joints are swollen and the involvement can be asymmetric sometimes; disabling acute tenosynovitis is frequently present.[4–8] Sometimes, atypical features such as Baker's cyst may be present. Sometimes sternoclavicular and temporomandibular joints may be involved; hips are relatively spared.[4,47,48] Swollen tender joints and crippling arthritis are usually evident [Figures 1 and 2] in almost all the patients at presentation. The pain may be severe enough to immobilize the patient and interfere with sleeping in the night. Joint pain may worsen with movement and back ache may also be present. Radiological findings are usually normal, and biological markers of inflammation may be normal or moderately elevated.[49,50]

### Effect on pregnancy

Chikungunya fever appears to have a direct impact on pregnancy with a higher risk of abortion in the first trimester and mother-to-child transmission in the last trimester.[4–8] In a study from the Reunion Islands outbreak, three out of nine miscarriages before 22 weeks of gestation were attributed to the Chikungunya virus infection documented by positive reverse transcription polymerase chain reaction (RT-PCR) in amniotic fluid.[51]

### Others

Several neurological, ocular, and hemorrhagic manifestations of fulminant hepatitis have been documented during the recent epidemic (*vide infra*).[52] Other uncommon manifestations include myocarditis after acute febrile illness,[53,54] mixed cryoglobulinemia in the first months after disease onset, induction for false-negative serologic tests for Chikungunya fever, and long persistence.[4]

## Course of the Disease

The fever is usually of short duration and usually resolves in 3 to 4 days. In some patients, a biphasic pattern of fever has been described with a febrile episode of 4 to 6 days, followed by a fever-free period of a few days followed by recurrence of fever (usually 101-102 °F) that may last a few days.

### Chronic stage

In a majority of the patients, the joint pains resolve in 1 to 3 weeks. However, the arthritis can persist in about 33% of patients for 4 months, 15% for 20 months, and in 12% for 3-5 years.[7,49,50,55] The chronic stage is characterized by unpredictable relapses that include sensation of fever, asthenia, and exacerbation of arthralgias and stiffness. Affected patients may manifest inflammatory polyarthritis, severe subacute tenosynovitis/bursitis (consequently nerve tunnel syndromes) in hands, wrists, and exacerbation of pain on movement in previously injured joints.[4,47] Older individuals and those with underlying rheumatic and traumatic joint disorders seem to be more vulnerable to develop the chronic stage.[4–8] Rarely, rheumatic manifestations resulting in joint destruction before resolution after 15 years have been reported.[56]

Some studies have documented occurrence of rheumatoid arthritis following Chikungunya fever, suggesting that the viral infection may have a role in the initiation or unmasking of rheumatoid arthritis.[57] This relationship merits further study.

## Chikungunya Fever in Neonates, Infants, and Children

### Neonates

Mothers afflicted with Chikungunya fever in the perinatal period (−4 days to +1 days) can transmit Chikungunya fever to neonates by vertical transmission.[58] Intrapartum transmission also contributes; caesarean section does not appear to prevent the transmission.[4,58,59] Neonatal Chikungunya fever is associated with fever, poor feeding, pain, distal edema, various skin manifestations, seizures, meningoencephalitis, and echocardiographic abnormalities in the newborn.[4,58]

### Infants

Chikungunya fever in infants manifests with certain differences.[58] Fever is commonly present; associated constitutional symptoms include lethargy or irritability and excessive cry. Acrocyanosis may be prominent; symmetrical superficial vesicobullous lesions, erythematous asymmetrical macules, and patches, which later progressed to morbiliform rashes, have also been described. The face and oral cavity are usually spared.[58,60]

### Children

Chikungunya fever in children resembles that observed in adults with important differences.[58] Common clinical manifestations include abrupt onset of high-grade fever, skin rashes, minor hemorrhagic manifestations, arthralgia/arthritis, lymphadenopathy, conjunctival injection, swelling of eyelids, and pharyngitis. Rare clinical features include neurological manifestations including seizures, altered level of consciousness, blindness due to retrobulbar neuritis, and acute flaccid paralysis. Rheumatological manifestations are somewhat less frequent in children. Pediatric subjects may also experience febrile seizures, vomiting, abdominal pain, and constipation.[58,61]

## Cardinal Features Observed in the Current Epidemic in India

The demographic characteristics and clinical presentation of Chikungunya fever documented during the current epidemic from India are listed in Table 2.[1,62–65] Majority of the patients had presented with fever, arthralgias, arthritis, and mucocutaneous manifestations. In addition to these series,[1,62–65] several rare manifestations have also been documented in other publications from India and are described below.

**Table 2:** Demographic characteristics and clinical presentation of Chikungunya fever in studies from India during the current epidemic

Variable	Study (reference)				
	
	Mohan (1) (*n* = 1226)*	Lakshmi *et al.* (62) (*n* = 296)	Suryawanshi *et al.* (63) (*n* = 405)	Kannan *et al.* (64) (*n* = 354)	Bandyopadhyay *et al.* (65) (*n* = 321)
Period of study	January 2006-July 2009*	March-December 2006	July-September 2006	2007	August-December 2007
Place of study	Tirupati, Andhra Pradesh	Hyderabad, Andhra Pradesh	Nagpur, Maharashtra	Four severely affected districts of Kerala (Pathanamthitta, Idukki, Kottayam and Thrissur)	Nine districts of West Bengal
Method of diagnosis	IgM antibodies positive (*n* = 914)	RT-PCR (*n* = 144); RT-LAMP (*n* = 20)	IgM antibodies positive (*n* = 87)	Community based survey	IgM antibodies positive
Age	Mean age ± SD (years) = 38.4 ± 18.2	Most affected age group = 31-40 years (34%)	Mean age ± SD (years) = 26 ± 11.7†	Most affected age group = 16-35 years (31%)	Most affected age group = 31-50 years (43%)
Male:Female	1.1:1	1:1.6	2.3:1†	1.2:1	2:1
Symptoms‡					
Fever	100	100	100	100	100§
Headache	64	31	56	98	70§
Chills	30	ND	ND	ND	55§
Arthralgias	98	100	100	99	96§
Myalgias	96	ND	ND	99	80§
Photophobia	11	ND	ND	ND	25§
Nausea	42	ND	ND	83	38§
Vomiting	16	04	ND	11	06§
Eye pain	ND	08	ND	12	ND
Physical signs‡					
Conjunctival suffusion	74	ND	ND	08	ND
Painful swollen joints	70	40	24	ND	16§
Lymphadenopathy	04	ND	04	ND	65§
Skin rash	08	28	16	81	02§
Oral ulcers	ND	ND	10	18	ND
Altered consciousness	01	ND	03	ND	ND
Bleeding manifestations	02	ND	01	01	04§
Others	High-grade fever (> 40 °C); (24%) fulminant hepatitis (02%)	Back pain 42%; shoulder pain 19%; difficulty in walking 06%; polyarthritis 12%	Transverse myelitis (*n* = 05); acute inflammatory demyelinating polyneuropathy (*n* = 02)	Edema (61.3%); distaste (86.4%)	Cough 05%; diarrhoea 04%; abdominal pain 03%; chest pain 03%; oedema of legs 03%‡

* Data updated from Reference 1;

† Described for confirmed cases;

‡ Percentage positive;

§ Described in 100 serologically confirmed patients; IgM: Immunoglobulin M; RT-LAMP: Real-time loop-mediated isothermal amplification; RT-PCR: Reverse transcription polymerase chain reaction; ND: Not described

### Rare manifestations

#### Neurological manifestations

Although described in other alphavirus infections, neurovirulence and neuroinvasiveness are not common manifestations of Chikungunya fever.[4–8] During the present epidemic, several neurological manifestations were documented. Wadia[66] described the following neurological manifestations reported in 359 patients seen at 5 centers: encephalitis (*n* = 175); neuropathy (*n* = 129); myelitis (*n* = 69); entrapment neuropathy (*n* = 34); and muscle injury described in 34 of 229 patients. In another Indian report from Kota (Rajasthan) from August to October 2006 (*n* = 20),[67] altered mental functions, seizures, focal neurological deficit with abnormal computed tomography of head and altered cerebrospinal fluid (CSF) biochemistry, and permanent neurological sequelae have been described. In a study from Nagpur,[68] of the 300 patients with Chikungunya fever seen during the June-December 2006, 49 (16.3%; 42 males) had neurological complications. These included encephalitis (*n* = 27; predominantly demyelinating type), myelopathy (*n* = 7), peripheral neuropathy (*n* = 7), myeloneuropathy (*n* = 7), and myopathy (*n* = 1). In another recent publication,[69] neurologic syndromes in 99 cases from Ahmedabad and Pune seen during 2006 included encephalitis (*n* = 57), encephalopathy (*n* = 42), and myelopathy (*n* = 14), or myeloneuropathy (*n* = 12). The report from Andaman Nicobar Islands[70] documented acute flaccid paralysis in four patients with Chikungunya fever.

### Ocular manifestations

Nodular episcleritis, acute iridocyclitis, uveitis, and neuroretinitis have been documented as unusual ocular manifestations of Chikungunya fever.[71–73]

### Hemorrhagic manifestations

Unlike dengue fever, hemorhhagic manifestations are uncommon in Chikungunya fever. When present, they are mild and more frequently encountered in Asian compared with African patients.[4–8] When present, these manifestations include epistaxis, bleeding from the gums, positive Hess test, subconjunctival bleed, and petechial/purpuric rash.

### Others

Sudden sensorineural hearing loss[74] and hypokalemic periodic paralysis[75] are other rare manifestations that have been reported. Severe systemic disease with multiple organ system involvement has also been observed during the recent epidemic.[69]

## Economic Impact

In India, the national burden of Chikungunya was estimated to be 25,588 disability adjusted life years (DALYs) lost during 2006 epidemic, with an overall burden of 45.26 DALYs per million (range 0.01 to 265.62 per million in different states). Persistent arthralgia was found to account for 69% of the total DALYs. The productivity loss in terms of income foregone was estimated to be a minimum of Rs. 391 million. The actual loss is thought to be substantially higher than these estimates.[9]

## Differential Diagnosis

Various conditions from which Chikungunya fever must be distinguished are listed in Table 3. Twin outbreaks of dengue fever and Chikungunya fever are known to occur frequently.[40] While it is virtually impossible to distinguish one of these conditions from another on clinical grounds, observations published by astute clinicians can be of some help. In a study published from Thailand,[76] it was reported that compared with patients with dengue hemorrhagic fever, subjects with Chikungunya fever were more likely to manifest arthralgia/arthritis, maculopaular rash, and conjunctival injection. However, laboratory testing is essential to distinguish Chikungunya fever from the other conditions.

**Table 3:** Differential diagnosis of Chikungunya fever

Other viral fevers
Dengue fever
West Nile fever
O'nyong-nyong fever
Ross river fever
Sindbis fever
Crimean-Congo fever
Bussuquara fever
Mayaro fever
Ebola fever
Hanta virus infection
Kyasanur forest disease
Lassa fever
Rubella
Parvovirus B19
Hepatitis B
Mumps
Herpesviruses
Parasitic infections
Falciparum malaria
Bacterial infections
Leptospirosis

## Diagnosis

Efforts should be directed to carry out the relevant laboratory tests to rule out other mimics [Table 3] and rule in Chikungunya fever. The gold standard for the diagnosis of Chikungunya fever is viral culture based on inoculation of mosquito cell cultures, mosquitoes, mammalian cell cultures, or mice.[4–8] However, viral culture is seldom done in routine clinical practice as these facilities are not widely available in India. It has the advantage of detecting a wide range of viruses. RT-PCR, real-time loop-mediated isothermal amplification (RT-LAMP)[62] have also been found to be useful molecular tools for the rapid diagnosis.[4–8,38,77] More frequently, serodiagnostic methods for the detection of immunoglobulin M (IgM) and immunoglobulin G (IgG) antibodies against Chikungunya virus in acute and convalescent sera are used. These include enzyme-linked immunosorbent assay (ELISA), indirect immunofluorescent method, hemagglutination inhibition, or neutralization techniques.[4–8,38] The IgM antibodies are detectable after a mean period of 2 days by ELISA immunofluorescent assay and they persist for periods ranging from several weeks to 3 months.[7] The IgG antibodies can be detected in convalescent samples and persists for years. Instances of persistence of IgM antibodies 18 months after disease onset in about 40% of symptomatic patients has also been described.[4,78,79] Thus, these tests appear to be poorly standardized and should be interpreted with caution. Diagnostic criteria for Chikungunya fever are listed in Table 4.[80,81]

**Table 4:** Diagnostic criteria for Chikungunya fever

Suspected case
A patient presenting with acute onset of fever usually with chills/rigors, which lasts for 3-5 days with multiple joint pains/swelling of extremities that may continue for weeks to months
Probable case
A *suspected case* (see above) with any one of the following:
History of travel or residence in areas reporting outbreaks
Ability to exclude malaria, dengue and any other known cause for fever with joint pains
Confirmed case
Any patient who meets one or more of the following findings irrespective of the clinical presentation
Virus isolation in cell culture or animal inoculations from acute phase sera
Presence of viral RNA in acute phase sera by RT-PCR
Presence of virus-specific IgM antibodies in single serum sample in acute or convalescent stage
Fourfold increase in virus-specific IgG antibody titer in samples collected at least three weeks apart

RNA: Ribonucleic acid; RT-PCR: Reverse transcription polymerase chain reaction; IgM: Immunoglobulin M; IgG: Immunoglobulin G

## Management

Treatment of Chikungunya fever is symptomatic and supportive. Adequate fluid intake must be ensured. Paracetamol or nonsteroidal anti-inflammatory drugs (NSAIDS) may be used for symptom relief. Aspirin should be avoided due to its effect on platelets. In a recently conducted double-blind, placebo-controlled, randomized trial[82] assessing the efficacy of chloroquine in patients with acute Chikungunya fever, 27 patients received chloroquine [600 mg (one dose) on day 1; 600 mg (administered as 300 mg twice daily) on days 2 and 3; and 300 mg on days 4 and 5; total dose 2400 mg]; and 27 patients received a placebo treatment. The authors[82] reported that there was no significant difference between groups regarding the duration of fever, arthralgia, or the decrease of viremia between the first and third days. However, at day 200, patients who received chloroquine complained more frequently of arthralgia than those who received placebo (*P* < 0.01). The authors[82] suggested that there was no justification for the use of chloroquine to treat acute Chikungunya infection.

Published evidence does not support the use of corticosteroids, antibiotics, or antiviral drugs in the management of Chikungunya fever, and indiscriminate use of these agents can be hazardous. Electrolyte imbalance, pre-renal acute renal failure, and bleeding manifestations should be watched carefully and managed accordingly.[1]

The optimal treatment strategy for viral arthropathy of Chikungunya fever is not known. In an open labeled pilot trial (n=20) that was published 25 years ago, Brighton[83] reported the efficacy of chloroquine phosphate (250 mg/day) in the treatment of patients with chronic Chikungunya arthritis. Of the 10 patients who completed 20 weeks of therapy, Ritchie articular index and morning stiffness improved significantly. Seven of the 10 patients reported improvement in their condition as per their assessment, while, 5 of the 10 had improved as per the evaluating clinician's assesment.[83] Further randomized controlled studies with a large sample size are required to clarify the role of chloroquine/hydroxychloroquine in the treatment of Chikungunya arthropathy.

## Mortality

Observations from the earlier epidemics including the experience from India till mid-1970s indicate that Chikungunya fever is a self-limiting disease; mortality was not documented. The official figures in the current release[43] and earlier releases by the Ministry of Health and Family Welfare, Government of India,[1,2,43] also do not document mortality due to Chikungunya. None of the patients with Chikungunya fever seen by us had died. However, there have been reports of mortality due to Chikungunya fever during the present epidemic in some publications.[44,66,84,85] Mavalankar *et al*.[85] compared the mortality rates in 2006 with those in 2002-2005 for Ahmedabad and found that 2,944 excess deaths occurred during the Chikungunya epidemic (August-November 2006) when compared with the average number of deaths in the same months during the preceding 4 years. These authors[85] suggested that the excess deaths were attributable to this epidemic.

Indiscriminate use of corticosteroids, NSAIDS (especially aspirin), and antibiotics can contribute to thrombocytopenia, gastrointestinal bleeding, nausea, vomiting, and gastritis. This may lead to dehydration, pre-renal acute renal failure, dyselectrolytemia, and sometimes hypoglycemia. These can indirectly contribute to the mortality due to Chikungunya fever.[1]

## Prevention

Patients with Chikungunya fever should be advised to avoid being bitten by mosquitoes as the disease can be transmitted to others. Presently no commercial vaccine is available for Chikungunya fever in India, although some candidate vaccines are being tested in human beings.[86,87]

### Vector surveillance and control

Educating the community and public health officials, vector control measures such as elimination of breeding sites and spraying of insecticides should be initiated at the individual and community levels as this can be rewarding. Vector surveillance and control is a key element in containing Chikungunya fever epidemics. Active involvement of community and public health authorities with regard to hygiene and mosquito control measures is essential to stand a chance in the war against the mosquitoes. Integrated vector management measures to reduce or interrupt transmission of disease must be pursued. These details are beyond the scope of this review and the reader is referred to Reference 42 for details.

## Future Directions

Chikungunya fever is emerging as a global disease. Several issues related to Chikungunya fever, such as, the reason(s) for the mysterious behavior of dramatic outbreaks interspersed by periods of prolonged absence; virus survival in nature and factors triggering outbreaks; reasons for ECSA strain replacing the Asian strain during the current epidemic, among others, need to be further studied. The quest for an effective vaccine is still on.
